# Put3 Positively Regulates Proline Utilization in *Candida albicans*

**DOI:** 10.1128/mSphere.00354-17

**Published:** 2017-12-13

**Authors:** Walters Aji Tebung, Raha Parvizi Omran, Debra L. Fulton, Joachim Morschhäuser, Malcolm Whiteway

**Affiliations:** a Chemistry and Biochemistry Department, Concordia University, Montreal, Quebec, Canada; b Institut für Molekulare Infektionsbiologie, Universität Würzburg, Würzburg, Germany; c Biology Department, Concordia University, Montreal, Quebec, Canada; Carnegie Mellon University

**Keywords:** *Candida albicans*, Put3, Put3 regulation, *Saccharomyces cerevisiae*, carbon source, nitrogen source, proline catabolism

## Abstract

The zinc cluster transcription factor Put3 was initially characterized in *Saccharomyces cerevisiae* as the transcriptional activator of *PUT1* and *PUT2*, two genes acting early in the proline assimilation pathway. We have used phenotypic studies, transcription profiling, and chromatin immunoprecipitation with microarray technology (ChIP-chip) to establish that unlike *S. cerevisiae*, which only uses proline as a nitrogen source, *Candida albicans* can use proline as a nitrogen source, a carbon source, or a source of both nitrogen and carbon. However, a *C. albicans put3* null mutant cannot grow on proline, suggesting that as in *S. cerevisiae*, *C. albicans* Put3 (CaPut3) is required for proline catabolism, and because the *C. albicans put3* null mutant grew efficiently on glutamate as the sole carbon or nitrogen source, it appears that CaPut3 also regulates the early genes of the pathway. CaPut3 showed direct binding to the *CaPUT1* promoter, and both *PUT1* and *PUT2* were upregulated in response to proline addition in a Put3-dependent manner, as well as in a *C. albicans* strain expressing a hyperactive Put3. CaPut3 directs proline degradation even in the presence of a good nitrogen source such as ammonia, which contrasts with *S. cerevisiae* Put3 (ScPut3)-regulated proline catabolism, which only occurs in the absence of a rich nitrogen source. Thus, while overall proline regulatory circuitry differs between *S. cerevisiae* and *C. albicans*, the specific role of Put3 appears fundamentally conserved.

**IMPORTANCE** *Candida albicans* poses a significant threat to the lives of immunocompromised people. Historically, knowledge has been drawn from studies on *Saccharomyces cerevisiae* to understand the pathogen, and many *Candida albicans* genes are named after their *S. cerevisiae* orthologs. Direct studies on the pathogen have, however, revealed differences in the roles of some orthologous proteins in the two yeasts. We show that the Put3 transcription factor allows the pathogen to completely degrade proline to usable nitrogen and carbon by evading regulatory restrictions imposed on its *S. cerevisiae* ortholog, which mandates conditional use of proline only as a nitrogen source in the baker’s yeast. The ability of *Candida albicans* to freely obtain nutrients from multiple sources may help it thrive as a commensal and opportunistic pathogen.

## Abstract (precis)

*Candida albicans* poses a significant threat to the lives of immunocompromised people. Historically, knowledge has been drawn from studies on *Saccharomyces cerevisiae* to understand the pathogen, and many *Candida albicans* genes are named after their *S. cerevisiae* orthologs. Direct studies on the pathogen have, however, revealed differences in the roles of some orthologous proteins in the two yeasts. We show that the Put3 transcription factor allows the pathogen to completely degrade proline to usable nitrogen and carbon by evading regulatory restrictions imposed on its *S. cerevisiae* ortholog, which mandates conditional use of proline only as a nitrogen source in the baker’s yeast. The ability of *Candida albicans* to freely obtain nutrients from multiple sources may help it thrive as a commensal and opportunistic pathogen.

## INTRODUCTION

Every organism requires carbon and nitrogen for survival, but nutrient choices and nutrient assimilation mechanisms vary among species. For example, *Candida albicans* uses available galactose as a carbon source even in the presence of glucose, in contrast to *Saccharomyces cerevisiae*, which shuts down the galactose catabolic pathway and preferentially uses glucose as a carbon source when both galactose and glucose are available (1). Differences in this regulatory circuitry are correlated with an exchange in key transcription factors; Gal4 controls galactose metabolism in *S. cerevisiae* through regulation of the Leloir pathway genes (1, 2), while Rtg1 and Rtg3 regulate expression of the orthologous genes in *C. albicans* (3). *S. cerevisiae* has the ability to acquire carbon and nitrogen from various sources, but preferentially utilizes nutrients from available sources (1, 4). Such examples of Gal4 transcriptional rewiring and altered metabolic dynamics in *C. albicans* and *S. cerevisiae* highlight a need to be cautious when relying solely on orthologous gene identification to infer protein function and metabolic pathways.

We have studied the role of Put3 in *C. albicans*, and our findings suggest that Put3 function is largely conserved between *C. albicans* and *S. cerevisiae*. However, *C. albicans* can use proline as both a carbon source and nitrogen source, unlike *S. cerevisiae*, where Put3 only activates proline catabolism in the absence of a rich nitrogen source. The presence of a rich nitrogen source does not prevent *C. albicans* Put3 (CaPut3) from directing the breakdown of proline to acquire nitrogen and carbon for cell growth. Nitrogen catabolism has been shown to be hierarchical in *S. cerevisiae*; proline is not used as a nitrogen source in the presence of a more readily assimilated source such as ammonium sulfate (4). Furthermore, proline only serves as a nitrogen source in *S. cerevisiae*: it does not provide both carbon and nitrogen availability (5). In *S. cerevisiae*, proline functions as an inducer of Put3, and available nitrogen sources dictate the phosphorylation status of Put3 and fine-tune its activation of Put1 and Put2 (4), an alteration that appears to be bypassed in *C. albicans* since the pathogen can catabolize proline in medium containing ammonium sulfate. Our findings suggest that Put3 function is fundamentally preserved between *C. albicans* (CaPut3) and *S. cerevisiae* (ScPut3), although their proline catabolism circuits show significant differences.

## RESULTS

### The Put3 ortholog in *C. albicans*.

**FIG 1:**
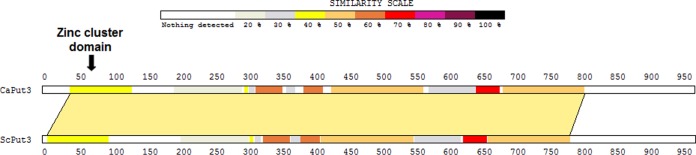
*C. albicans* Put3 aligned with *S. cerevisiae* Put3. The shaded alignment area has 41.7% identity and includes the zinc cluster domain.

In the pathogen *C. albicans*, the orf19.6203 (*PUT3*) gene encodes the proposed ortholog of *S. cerevisiae* Put3. The *S. cerevisiae* and *C. albicans* proteins have about 42% sequence identity spanning 821 amino acids (over 83% of each protein) at the N-terminal part of each ortholog (Fig. 1). The next closest *S. cerevisiae* protein homolog to CaPut3 is Asg1, which has a stretch of 214 amino acids with just 24.8% identity and does not include the zinc cluster domain. These findings suggest that the *C. albicans* orf19.6203 gene may be orthologous to the yeast *put3* gene.

### Proline utilization.

**FIG 2:**
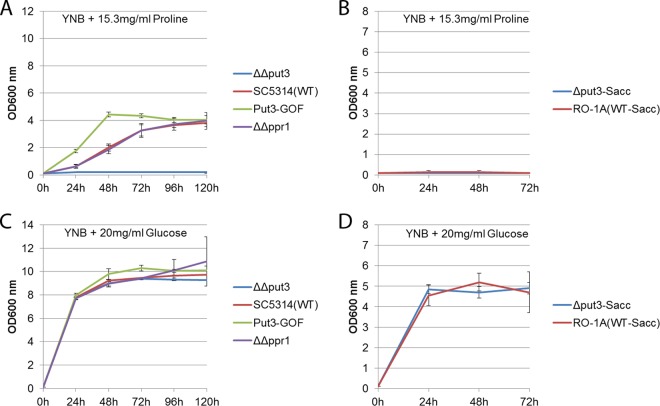
Proline utilization as a carbon source. Shown are the results from an assay of a *C. albicans put3* null mutant (ΔΔput3), the *C. albicans* wild-type (WT) strain, SC5314, a *C. albicans* Put3 gain-of-function mutant (Put3-GOF), the *C. albicans ppr1* null mutant (ΔΔppr1), the *S. cerevisiae put3* null mutant (Δput3-Sacc), and the *S. cerevisiae* wild-type strain, RO-1A (WT-Sacc), at 30°C. YNB plus glucose was used as a control at 30°C. (A) Growth curve of the *C. albicans* ΔΔput3 mutant, SC5314, the Put3-GOF mutant, and the ΔΔppr1 mutant in YNB with proline as the sole carbon source at 30°C. (B) Growth curve of the *S. cerevisiae* Δput3-Sacc mutant and RO-1A in YNB with proline as the sole carbon source at 30°C. (C) Growth curve of the *C. albicans* ΔΔput3 mutant, SC5314, the Put3-GOF mutant, and the ΔΔppr1 mutant in YNB with glucose as the sole carbon source at 30°C. (D) Growth curve of the *S. cerevisiae* Δput3-Sacc mutant and RO-1A in YNB with glucose as the sole carbon source at 30°C. Error bars are based on the standard deviation from two biological replicates of each data point reading taken every 24 h.

Phenotypic studies showed that the *C. albicans* wild-type (WT) strain SC5314 and the disruption *ppr1* null mutant strain grow in yeast nitrogen base medium with ammonium sulfate (YNB plus NH_4_^+^) that contains 15.3 mg/ml proline as the sole carbon source (Fig. 2A). These findings revealed that *C. albicans* is able to metabolize and use proline as the sole carbon source even in the presence of ammonium sulfate (Fig. 2B), an observation that contrasts with previous findings, further confirmed in this study (Fig. 2B), that *S. cerevisiae* cannot use proline as the sole carbon source (5). This suggests that the pathogen is able to make more liberal use of proline as a carbon source (Fig. 2A).

To confirm whether the *C. albicans* Put3 ortholog is a regulator of proline catabolism, we cultured the wild type as well as the *put3* null mutant and Put3 gain-of-function (Put-GOF) mutant strains for 5 days at 30°C in YNB medium containing 15.3 mg/ml proline as the sole carbon source. The *put3* null mutant strain did not grow in YNB with proline as the sole carbon source even after 5 days at 30°C, suggesting an inability of the strain to metabolize proline (Fig. 2A), while the Put3-GOF strain showed better growth than either the wild-type strain, SC5314, or the *ppr1* null mutant strain used as a control. Ppr1 is a different zinc cluster transcription factor functioning in purine catabolism (6); it serves as a control for the *put3* null mutant strain since it was constructed similarly (Fig. 2A). These findings suggest that Put3 regulates proline catabolism in *C. albicans*. All *C. albicans* strains had normal and similar growth patterns in YNB with 20 mg/ml glucose as the sole carbon source (Fig. 2C), as well as in YPD (yeast extract, peptone, dextrose), and all *C. albicans* strains showed no growth in YNB without any carbon source (data not shown). As expected, during growth under the same experimental conditions for 3 days, both the *S. cerevisiae* wild-type strain, RO-1A, and the *S. cerevisiae put3* null mutant strain had similar growth outcomes in YNB with 20 mg/ml glucose as the sole carbon source (Fig. 2D) and no growth in YNB with 15.3 mg/ml proline as the sole carbon source (Fig. 2B).

**FIG 3:**
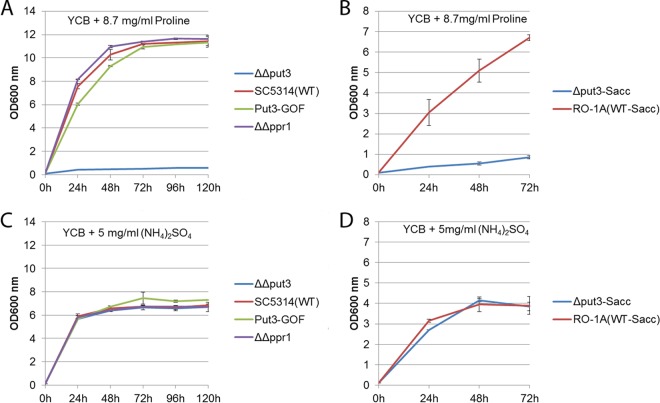
Proline utilization as a nitrogen source. Shown are the results from an assay of a *C. albicans put3* null mutant (ΔΔput3), the *C. albicans* wild-type (WT) strain, SC5314, a *C. albicans* Put3 gain-of-function mutant (Put3-GOF), the *C. albicans ppr1* null mutant (ΔΔppr1), the *S. cerevisiae put3* null mutant (Δput3-Sacc), and the *S. cerevisiae* wild-type strain, RO-1A (WT-Sacc), at 30°C. YCB plus ammonium sulfate was used as a control at 30°C. (A) Growth curve of the *C. albicans* ΔΔput3 mutant, SC5314, the Put3-GOF mutant, and the ΔΔppr1 mutant in yeast carbon base (YCB) with proline as the sole nitrogen source at 30°C. (B) Growth curve of the *S. cerevisiae* Δput3-Sacc mutant and RO-1A in YCB with proline as the sole nitrogen source at 30°C. (C) Growth curve of the *C. albicans* ΔΔput3 mutant, SC5314, the Put3-GOF mutant, and the ΔΔppr1 mutant in YCB with ammonium sulfate as the sole nitrogen source at 30°C. (D) Growth curve of the *S. cerevisiae* Δput3-Sacc mutant and RO-1A in YCB with ammonium sulfate as the sole nitrogen source at 30°C. Error bars are based on the standard deviation from two biological replicates of each data point reading taken every 24 h.

**FIG 4:**
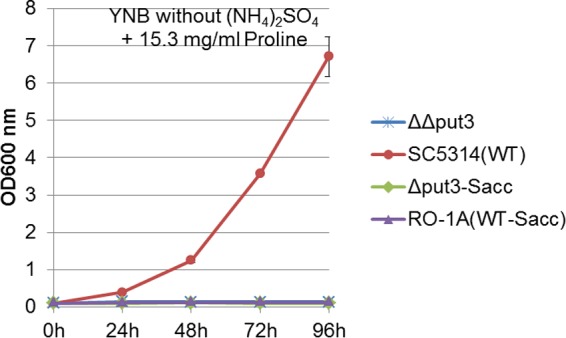
Proline utilization as a source of both carbon and nitrogen. Shown are the results from an assay of the *C. albicans put3* null mutant (ΔΔput3), the *C. albicans* wild-type (WT) strain, SC5314, the *S. cerevisiae put3* null mutant (Δput3-Sacc), and the *S. cerevisiae* wild-type strain, RO-1A (WT-Sacc), at 30°C. The growth curves of the *C. albicans* ΔΔput3 mutant and SC5314 and the *S. cerevisiae* Δput3-Sacc mutant and RO-1A in yeast nitrogen base (YNB) without ammonium sulfate but with proline as the sole carbon and nitrogen source at 30°C were determined. Error bars are based on the standard deviation from two biological replicates of each data point reading taken every 24 h.

We also used 8.7 mg/ml proline as the sole nitrogen source in yeast carbon base medium (YCB) to culture wild-type *C. albicans* strain SC5314, the control *ppr1* null mutant strain, the *put3* null mutant strain, and the Put3 gain-of-function strain. All strains were able to grow in the medium except the *put3* null mutant strain (Fig. 3A), suggesting that Put3 regulates the use of proline as both a carbon source and a nitrogen source in *C. albicans*. An *S. cerevisiae* prototrophic strain, RO-1A, was able to grow in 8.7 mg/ml proline as the sole nitrogen source, but the *S. cerevisiae put3* null mutant strain failed to grow in the same culture medium (Fig. 3B). All *C. albicans* strains showed similar growth in YCB medium with 5 mg/ml ammonium sulfate as the sole nitrogen source (Fig. 3C), and both the *S. cerevisiae* prototrophic strain RO-1A and the *S. cerevisiae put3* null mutant strain had similar growth patterns with 5 mg/ml ammonium sulfate as the sole nitrogen source (Fig. 3D). We further confirmed that the wild-type *C. albicans* strain SC5314 grows in YNB medium containing proline as the sole source of both carbon and nitrogen, but as previously noted (5), *S. cerevisiae* prototrophic strains such as RO-1A cannot use proline as a combined carbon and nitrogen source (Fig. 4) and can only use proline as a nitrogen source in the absence of a rich nitrogen source such as ammonium sulfate. The inability of both *S. cerevisiae* and *C. albicans put3* mutants to utilize proline as the sole nitrogen source confirms that Put3 is essential for proline catabolism in both species. Both the *S. cerevisiae* wild-type strain and the *S. cerevisiae put3* mutant strain also failed to use proline as the sole carbon source. This could be due to the inhibitory effect of ammonium sulfate present in the medium; however, both strains failed to utilize proline as the sole source of both nitrogen and carbon, even when other nitrogen sources were absent from the growth medium. The inability of *S. cerevisiae* to degrade proline for carbon use even in the absence of a nitrogen source agrees with a previous report that *S. cerevisiae* cannot utilize associated amino acids as a carbon source (7).

### Identification of proline-regulated genes.

To establish whether the presence of proline impacted *C. albicans* gene expression, we performed transcriptome sequencing (RNA-seq) analyses of *C. albicans* cells growing in the presence or absence of proline. As shown in Table 1, the addition of proline to cultures containing a readily accessible carbon (glucose) and nitrogen (ammonium) source dramatically activated the expression of *PUT1* and *PUT2*. We observed an even higher induction of *PUT1* and *PUT2* expression by proline when both ammonium sulfate and glucose were absent and when either ammonium sulfate or glucose was absent (Table 1). Expression of the Put3 transcription factor itself was not induced by proline, and its deletion eliminated the proline-induced expression of *PUT1* and *PUT2* (see Table S3 in the supplemental material). These findings suggest that proline induces the expression of *PUT1* and *PUT2* by interacting with Put3 at a posttranscriptional level. Ribosome biogenesis genes were also induced by proline addition when ammonium sulfate, glucose, or both were missing from the media (Table 1). When both ammonium sulfate and glucose were present, the induction of genes involved in ribosome biogenesis was not observed, and induction was lower when ammonium sulfate was missing and glucose was present in the media (Table 1). This could indicate that glucose represses, to a certain degree, the Put3-dependent induction of ribosome biogenesis by proline. Proline also induced genes encoding carboxylic acid catabolism and glyoxylate enzymes, such as *ICL1*, *FOX2*, and *ADH2*, when glucose, ammonium sulfate, or both were missing in the media. These genes are required by the pathogen to obtain carbon from nonfermentable carbon sources.

**TABLE 1:** Proline-induced genes in *C. albicans* SC5314

Gene or allele^a^	Fold change in expression^b^:			
	YNGP_SC/YNG_SC	YP_SC/YNG_SC	YNP_SC/YNG_SC	YGP_SC/YNG_SC
*PUT1*	47.05	202.58	238.72	215.67
*PUT2*	18.59	42.76	43.92	50.47
C2_02580W_A	2.65	14.21	10.61	12.57
*ICL1*	2.45	11.92	15.20	12.39
*FOX2*	1.97	8.21	9.86	7.97
*ADH2*	0.83	13.13	31.08	17.90
*BUD23*	1.02	15.52	13.79	8.68
C1_06760C_A,B	1.66	13.13	12.79	6.19
C1_10970W_A,B	0.84	7.77	9.25	3.18
C2_04570W_A,B	1.09	9.60	8.76	4.25
C3_02020W_A,B	1.53	11.84	9.94	6.35
C3_06760W_A,B	1.29	14.19	15.81	7.71
C5_04840C_A,B	1.78	11.16	13.42	5.90
*DRS1*	1.34	14.59	10.49	8.70
*HIT1*	0.00	6.85	12.08	4.79
*SDA1*	1.20	10.38	10.68	4.73
*SPB4*	1.15	8.80	10.61	4.87
*UTP21*	1.09	10.68	10.96	4.61
C1_02450C_A,B	1.07	5.73	7.97	3.33
C1_04040C_A,B	0.93	8.22	9.36	4.48
C2_05750W_A,B	0.54	5.86	8.67	3.71
*CHR1*	1.07	6.68	8.23	3.41
CR_03360W_A,B	0.92	6.50	8.23	3.24
CR_09800C_A,B	0.83	5.06	10.06	4.77
*REI1*	0.71	6.96	9.14	3.04
*RPP1*	0.43	7.12	10.72	4.52
*RRP8*	0.81	6.14	7.69	3.12
CR_01780W_A,B	1.73	9.20	3.60	4.62
CR_10410C_A,B	1.21	10.31	7.43	5.48
*FYV5*	0.71	8.15	5.67	4.11
*LTV1*	0.67	14.55	9.33	8.94
*NOP14*	0.82	10.75	8.97	5.70
*YVH1*	0.72	7.57	5.67	3.83

a RNA-seq data showing *C. albicans* SC5314 genes induced by proline are presented. The genes shown have at least one allele induced 10-fold or more under at least one of the conditions listed and are involved in either proline degradation, the glyoxylate cycle, or ribosome biogenesis. When the allele name is used instead of the gene name, “A,B” is added at the end of the name if the data presented are for both alleles of the gene.

b The average of the expression fold change for the two alleles of each gene is presented, except for C2_02580W_A, which is just one allele of the gene. The YNPG_SC/YNG_SC column shows the fold change in gene expression for the *C. albicans* SC5314 wild-type strain cultured in YNB containing ammonium sulfate, glucose, and proline compared against the same strain grown in YNB containing ammonium sulfate and glucose. The YP_SC/YNG_SC column shows the fold change in gene expression for the *C. albicans* SC5314 wild-type strain cultured in YNB containing proline only compared against the same strain grown in YNB containing ammonium sulfate and glucose. The YNP_SC/YNG_SC column shows the fold change in gene expression for the *C. albicans* SC5314 wild-type strain cultured in YNB containing ammonium sulfate and proline compared against the same strain grown in YNB containing ammonium sulfate and glucose. The YGP_SC/YNG_SC column shows the fold change in gene expression for the *C. albicans* SC5314 wild-type strain cultured in YNB containing glucose and proline compared against the same strain grown in YNB containing ammonium sulfate and glucose.

### Identification of Put3-regulated genes using transcriptional profiling.

To further confirm the pathway through which Put3 regulates proline catabolism in *C. albicans*, we performed transcriptional profiling experiments using the Put3 gain-of-function (Put3-GOF) mutant strain SCPUT3GAD1A generated by addition of a Gal4 activation domain to Put3 (8). Such GOF mutant strains can allow for gene network upregulation in the absence of any stimulatory condition. Transcription profiling using hyperactive proteins is an efficient way to identify the regulatory pathway of transcription factors (9–11). The key proline catabolism enzyme-encoding genes *PUT1* and *PUT2* (Table 2) were among the genes upregulated in strains expressing the hyperactive Put3. As well, the *C. albicans* gene orf19.1584, which is similar to the gene *MCH5* encoding a riboflavin transporter required for Put1 function in proline degradation in *S. cerevisiae* (12), was also upregulated. This suggests that Put3 has preserved its proline catabolism regulation role in *C. albicans* and *S. cerevisiae*, and it regulates this pathway by transcriptionally activating *PUT1* and *PUT2*, as well as the Put1 cofactor precursor importer gene, orf19.1584. Other upregulated genes observed in the transcription profiling data using the Put3 gain-of-function mutant strain include genes involved in ribosome biogenesis, carboxylic acid metabolic processes, filamentous growth, response to stress, RNA metabolic processes, and cellular protein modification (Table 2; see Table S2 in the supplemental material).

**TABLE 2:** *C. albicans* Put3-regulated genes and potential target genes

Gene	ORF	Gene categorization by^a^:			
		Transcription profiling	ChIP-chip	ScPut3 motif	ScPut3 regulates ortholog
	orf19.1584	✓	✓	✓	✓
*CYB2*	orf19.5000	✓	✓	✓	
	orf19.670.2	✓	✓	✓	
	orf19.3406	✓	✓	✓	
*YHB1*	orf19.3707	✓	✓	✓	
	orf19.1109	✓	✓		
*CAS1*	orf19.1135	✓	✓		
*FAD2*	orf19.118	✓	✓		
	orf19.1486	✓	✓		
	orf19.1549	✓	✓		
*UTP4*	orf19.1633	✓	✓		
	orf19.1789	✓	✓		
*RGT1*	orf19.2747	✓	✓		
	orf19.2782	✓	✓		
*AIP2*	orf19.300	✓	✓		
	orf19.3254	✓	✓		
	orf19.3585	✓	✓		
*IDP2*	orf19.3733	✓	✓		
	orf19.4273	✓	✓		
*PUT1*	orf19.4274	✓	✓		✓
*PPS1*	orf19.4405	✓	✓		
*PTC8*	orf19.4698	✓	✓		
	orf19.5026	✓	✓		
*RPL35*	orf19.5964.2	✓	✓		
*GLN1*	orf19.646	✓	✓		
*GIN4*	orf19.663	✓	✓		
	orf19.6828	✓	✓		
	orf19.6853	✓	✓		
*ATP1*	orf19.6854	✓	✓		
*CAN3*	orf19.84	✓	✓		
*PET9*	orf19.930	✓	✓		
	orf19.1054		✓	✓	
*PEX11*	orf19.1089		✓	✓	
	orf19.1490.1		✓	✓	
*HEM3*	orf19.1742		✓	✓	
	orf19.2105		✓	✓	
*TIF5*	orf19.4261		✓	✓	
*RAD9*	orf19.4275		✓	✓	
	orf19.6188		✓	✓	
	orf19.7266		✓	✓	
*MIS12*	orf19.7534		✓	✓	
*PUT2*	orf19.3974	✓			✓
*TEC1*	orf19.5908	✓			✓
*AXL1*	orf19.7342	✓			✓
*STE3*	orf19.2492		✓		✓
*PCL1*	orf19.2649		✓		✓
*KIP4*	orf19.5265		✓		✓
*BEM2*	orf19.6573		✓		✓
	orf19.68.2		✓		✓
*MODF*	orf19.5029			✓	✓
	orf19.5720			✓	✓

a Both ChIP-chip and transcriptional profiling data were analyzed using GenePix and MeV, and Put3 motif identification was carried out using the online motif scanning tool FIMO. *C. albicans* genes appearing in any two of the following four categories are presented: (i) gene is upregulated in transcription profiling data for SCPUTGAD1A, (ii) gene shows ChIP-chip binding by Put3, (iii) promoter has ScPut3 binding motif, and (iv) ortholog is regulated by ScPut3. Check marks indicate when a gene falls into a category. Transcription profiling median of ratio values above 1.94 and ChIP-chip log of ratio values above 1.4 are considered significant.

### Direct identification of genes bound by *Candida albicans* Put3 using ChIP-chip.

Our phenotypic studies demonstrated that Put3 regulates proline use in *C. albicans*, and transcription profiling experiments highlighted that *C. albicans* Put3 regulates the proline catabolism pathway genes *PUT1*, *PUT2*, and orf19.1584, as well as genes involved in ribosome biogenesis and other cellular pathways. Chromatin immunoprecipitation followed by microarray analysis (ChIP-chip) can identify the direct binding targets of specific transcription factors. This analysis was carried out using the *C. albicans* strain SCPUT3GAD1A (8), which was constructed from SC5314 and contains a hemagglutinin (HA) epitope sequence fused to the Put3 protein. After chromatin cross-linking, target binding sequences were identified by amplifying and labeling immunoprecipitated DNA sequences and hybridizing these labeled sequences to Agilent 8X15K whole-genome tiling arrays containing a representative probe set for the *C. albicans* genome. Put3 target genes were ranked based on their log of ratio (PUT3-HA-Cy5 versus nontagged Cy3) values (Table S2), and then target genes with log of ratio values of at least 1.4 that were also upregulated in the SCPUT3GAD1A strain expressing a hyperactive Put3 (8) (Table 2) were further annotated for function using the Candida Genome Database Gene Ontology tool. Consistent with transcription profiling data for the SCPUT3GAD1A strain, Put3 targets identified by ChIP-chip include the promoters of *PUT1* and orf19.1584, as well as genes involved in ribosome biogenesis, carboxylic acid metabolic processes, filamentous growth, response to stress, RNA metabolic processes, and cellular protein modification (Table 2). *PUT2*, however, was not significantly bound by *C. albicans* Put3 in our ChIP-chip data; this suggests that Put3 may regulate *PUT2* indirectly. Binding of *C. albicans* Put3 to the promoter of *PUT1* and orf19.1584 provides further support for Put3’s regulatory role in proline catabolism in *C. albicans*, which aligns with the role played by its ortholog in *S. cerevisiae*. No classic Put3 DNA binding motif as defined in *S. cerevisiae* can be identified at the *C. albicans PUT1* promoter. The orf19.1584 promoter, however, has a sequence (*P* = 6.89E−6) identical to the predicted *S. cerevisiae* Put3 (ScPut3) binding motif (13) located at positions −42 to −57. Other genes with minimum log ratios of 1.4 identified as both upregulated in the gain-of-function strain and with demonstrated ChIP-chip binding were also assessed for the *S. cerevisiae* Put3 binding motif. In addition to orf19.1584, four other genes (orf19.5000, orf19.670.2, orf19.3406, and orf19.3707) in this set of 31 genes had a predicted *S. cerevisiae* Put3 motif. Overall, however, this motif is relatively common in the *C. albicans* intergenic regions, with 668 sequences identified in the 6,206 *C. albicans* promoter sequences used by the FIMO motif scan tool (14). Thus, the predicted *S. cerevisiae* Put3 motif is not significantly enriched at the promoters of genes present in both our ChIP-chip and transcription profiling hits. However, no alternative putative motif was detected within the sequences of the Put3-bound and transcriptionally activated genes in *C. albicans*.

### Conservation of Put3 phosphorylation sites.

**FIG 5:**
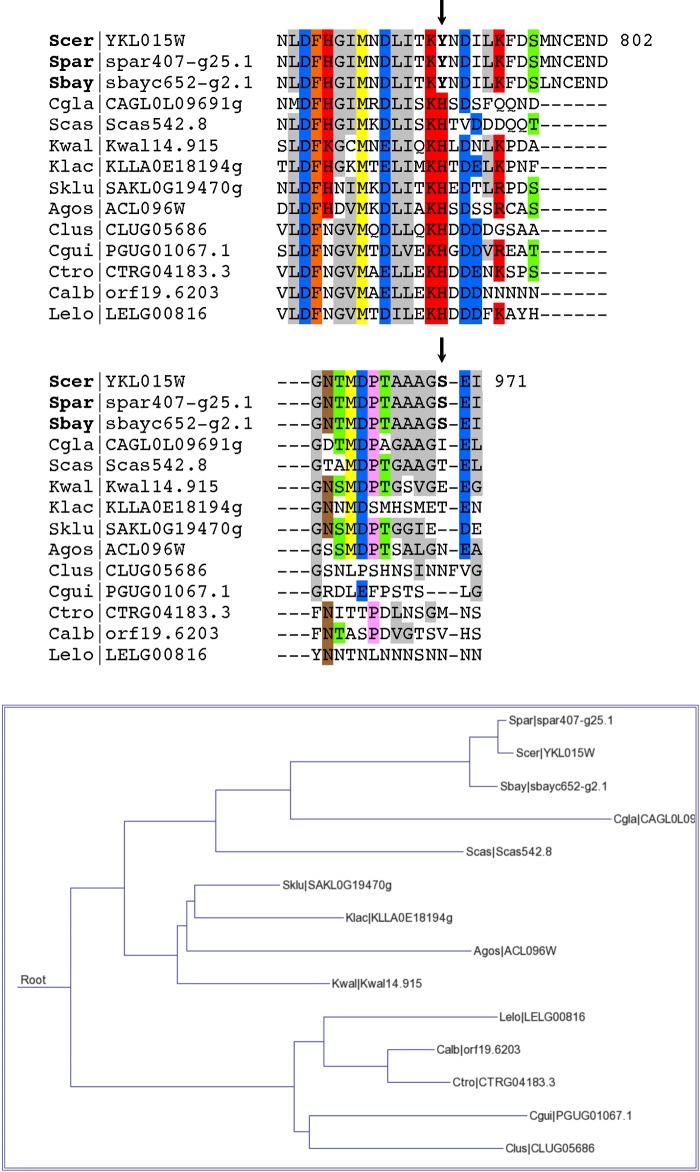
Alignment of Put3 through yeast phylogeny. Shown is alignment of Put3 and orthologs through yeast phylogeny. A conserved tyrosine essential for Put3 activation and conserved serine essential for Put3 inhibition in *S. cerevisiae* are in boldface, and boldface arrows indicate their positions in the aligned sequences. Species name abbreviations with the conserved activating tyrosine and inhibiting serine are in boldface: Scer, *Saccharomyces cerevisiae*; Spar, *Saccharomyces paradoxus*; Sbay, *Saccharomyces bayanus*. Amino acids of the same class that are 50% or more conserved are highlighted in specific colors based on amino acid class. A phylogenic tree shows Put3 phylogeny in yeasts.

Studies in *S. cerevisiae* have shown that Put3 regulation depends on various factors, including proline and available nitrogen sources. The presence of proline alters the conformation of Put3 to a more active form (15): meanwhile, different nitrogen sources induce specific Put3 phosphorylation states that range from an active state to a less active state as the quality of the nitrogen source increases (4, 16). Phosphorylation of Y788 activates Put3, and phosphorylation of S969 inhibits Put3 (17). Put3 alignment among fungal species shows that the phosphorylatable Y788 and S969 both appear in the lineage leading to *Saccharomyces bayanus* and are conserved through *Saccharomyces cerevisiae*. Y788 and S969 are not present in species appearing earlier than *Saccharomyces bayanus* in the phylogeny, including *C. albicans* (Fig. 5). These results suggest that Put3 may have acquired its potential to be regulated by these phosphorylations at the lineage leading to *S. bayanus* and the potential has been conserved through *S. cerevisiae*.

## DISCUSSION

Put3 transcriptionally regulates enzymes of the proline catabolism pathway in *S. cerevisiae*. The *C. albicans* gene orf19.6203 has been named *PUT3* based on sequence similarity to *S. cerevisiae Put3*, but zinc cluster transcription factor-rewiring events such as those for Gal4 and Ppr1 (2, 6) highlight the need to study each transcription factor beyond sequence and structural alignments in order to accurately establish the role of each ortholog in other species. We performed phenotypic studies using a *C. albicans put3* null mutant and found that this mutant failed to grow in YNB medium containing proline as the sole carbon source (Fig. 2A) or in YCB medium containing proline as the sole nitrogen source (Fig. 3A), suggesting Put3 does play a role in proline catabolism in this opportunistic pathogen. In contrast, the wild-type strain SC5314, the Put3-GOF strain, and the *ppr1* null mutant strain used as controls all showed significant growth in both YNB medium containing proline as the sole carbon source (Fig. 2A) and YCB medium containing proline as the sole nitrogen source (Fig. 3A). Intriguingly, the Put3-GOF strain had a shorter lag phase in YNB medium with proline as the sole carbon source, which further suggests the importance of Put3 in proline catabolism (Fig. 2A). All strains showed similar growth in YNB medium containing glucose as the sole carbon source (Fig. 2C), in YCB medium containing ammonium sulfate as the sole nitrogen source (Fig. 3C), and in YPD medium.

Put3 regulates proline use as a nitrogen source in *S. cerevisiae* by controlling expression of *PUT1* and *PUT2*, which encode the factors required to process proline to glutamate. We found that *C. albicans put3* null mutant strain utilizes glutamate for both its carbon and nitrogen needs, which led us to hypothesize that Put3 could direct expression of the *PUT1* and *PUT2* genes for proline utilization. This prediction was confirmed using RNA-seq; in *C. albicans*, proline induces *PUT1* and *PUT2* transcription in a Put3-dependent manner (Table 1) (4). Furthermore, transcription profiling of an activated Put3 protein and ChIP-chip data support the role of Put3 in the positive regulation of *PUT1* and *PUT2*. This suggests that the zinc cluster transcription factor Put3 has conserved its role in proline catabolism between *C. albicans* and *S. cerevisiae*. Our data suggest that proline induces *PUT1* and *PUT2* by acting on Put3 at a posttranscriptional level since proline did not induce *PUT1* and *PUT2* in *put3* null mutant strains, and only *PUT1* (approximately 50-fold) and *PUT2* (approximately 20-fold), but not *PUT3*, were transcriptionally induced when both ammonium sulfate and glucose were present. In fact, *PUT1* ranked first and *PUT2* ranked second in our list of most upregulated genes upon proline induction in the presence of ammonium sulfate and glucose (Table 1). The inducing effect of proline was significantly increased in the absence of both ammonium sulfate and glucose, with *PUT1* induced approximately 200-fold and *PUT2* induced approximately 40-fold (Table 1). These findings suggest that *C. albicans* simultaneously uses proline, ammonium sulfate, and glucose when all are available in the media, but further induces Put3 upregulation of *PUT1* and *PUT2* expression when proline becomes the only source of carbon, nitrogen, or both carbon and nitrogen, as this may allow more efficient breakdown of proline to usable carbon and nitrogen for cellular needs. Although *PUT1* and *PUT2* have greater expression when proline is the sole source of carbon, nitrogen, or both, sufficient expression is still attained in the presence of glucose, ammonium sulfate, or both to degrade proline for cellular growth. Proline also induced the expression of genes involved in ribosome biogenesis when ammonium sulfate, glucose, or both were missing in the media. Carboxylic acid catabolism and glyoxylate pathway enzymes such as *ICL1*, *FOX2*, and *ADH2* were also upregulated by proline, but only when glucose, ammonium sulfate, or both were missing in the media. The induction of these enzymes could act to allow *C. albicans* to efficiently utilize proline as a carbon source. It is possible that the presence of ammonium sulfate and glucose reduces the need for the cells to break down available proline, which circumvents upregulation of carboxylic acid catabolism and glyoxylate enzymes. Overall, *C. albicans* is able to use proline as the sole source of carbon (Fig. 2A), nitrogen (Fig. 3A), and both carbon and nitrogen (Fig. 4). This is in clear contrast to *S. cerevisiae*, which can use proline only as a nitrogen source and does so only in the absence of a more readily assimilated source (5) (Fig. 2B, 3B, and 4). Notably, *C. albicans* can metabolize proline even in the presence of ammonium sulfate (Fig. 2A).

This nitrogen-sensing regulation of *S. cerevisiae PUT1* and *PUT2* by Put3 is achieved at least in part by fine-tuning Put3 activity through phosphorylation events that depend on the quality of available nitrogen in the growth medium (4, 16). Rich nitrogen sources induce phosphorylations that block the ability of Put3 to induce genes involved in proline catabolism. Sequence alignment of *S. cerevisiae* and *C. albicans* Put3 ortholog sequences revealed that the amino acids Y788 and S969, which are essential phosphorylation targets for *S. cerevisiae* Put3 activation and inhibition, respectively, are not conserved in *C. albicans* Put3 (17). This finding suggests that in *C. albicans*, Put3 (CaPut3) may not be equivalently regulated by phosphorylation in response to the quality of available nitrogen sources and may be compensated by the capacity of the pathogen to utilize proline as a carbon source in the presence of ammonium sulfate. Similar to *C. albicans*, other species appearing prior to *S. bayanus* in the yeast phylogeny lack these key amino acids implicated in Put3 regulation by phosphorylation.

While one might anticipate that differing phosphorylation states could reduce the capacity of *S. cerevisiae* to utilize proline as a nitrogen source in the presence of richer nitrogen sources, our study highlighted its complete inability to utilize proline when richer nitrogen sources are absent. This inability may be due in part to the role of the Fmp12 protein. Recent studies (7) have shown that the Fmp12 protein in *S. cerevisiae* has sequence similarity to α-ketoglutarate-dependent dioxygenases of *Candida* species and in humans plays a role in the decarboxylation of α-ketoglutarate, an intermediate of proline catabolism. The Fmp12-dependent decarboxylation of α-ketoglutarate might enable the bypass of the tricarboxylic acid (TCA) cycle reactions mediated by α-ketoglutarate dehydrogenase (KGDH) and succinyl coenzyme A (succinyl-CoA) ligase. As such, in *S. cerevisiae* α-ketoglutarate could be metabolized through Fmp12 rather than KGDH, allowing the bypass of NADH production via KGDH and ATP production via succinyl-CoA ligase (7). Overall, this would result in the inability of *S. cerevisiae* to generate sustainable energy for cell growth using proline as the sole source of carbon or both carbon and nitrogen. Intriguingly, deletion of *FMP12* promotes the use of proline as the sole source of both nitrogen and carbon by *S. cerevisiae*, and this enhanced-growth phenotype is eliminated by deletion of *PUT1* or *PUT2* (7). On the other hand, overexpression of *FMP12* negatively affected *S. cerevisiae* growth in media containing proline as the sole source of both nitrogen and carbon (7). If the α-ketoglutarate-dependent dioxygenases in *C. albicans*, such as Bbh1 (the ortholog of Fmp12), have a lower affinity for α-ketoglutarate compared to KGDH, this may explain the differences observed in the use of proline as a carbon and nitrogen source in the two species.

We further confirmed the role of Put3 in *C. albicans* using transcriptional profiling and ChIP-chip experiments. Consistent with our observations from phenotypic studies and RNA-seq data, both *PUT1* and *PUT2* in *C. albicans* are under the regulation of Put3, as shown by our transcriptional profiling data, and our ChIP-chip data suggest that Put3 binds the *PUT1* promoter (Table 2), while *PUT2* does not appear to be bound by Put3 in *C. albicans*. *MCH5* is also a direct target of Put3 in *S. cerevisiae* and encodes a riboflavin transporter (12). Riboflavin is required for the generation of flavin adenine dinucleotide (FAD), the catalytic cofactor required for Put1 activity (12). We discovered in *C. albicans* that one of the four genes with similarity to *MCH5*, orf19.1584, has the *S. cerevisiae* Put3 binding site CGG(N_10_)CCG (18) at its promoter, demonstrated direct binding by Put3, and is transcriptionally activated by Put3 (Table 2), suggesting that orf19.1584 is the functional ortholog of *MCH5*. orf19.1584, like its *S. cerevisiae* ortholog, could therefore play a role in the proline degradation pathway by importing riboflavin in *C. albicans* under the regulation of Put3. As shown in our RNA-seq data (Table 1), orf19.1584 (C2_02580W_A) was also upregulated by proline when ammonium sulfate, glucose, or both were missing in the culture media. Some *S. cerevisiae* Put3 targets (including Put1 and Put2) that have the Put3 binding site CGG(N_10_)CCG (18) at their promoters have clear orthologs in *C. albicans*. We, however, did not identify a predicted *S. cerevisiae* Put3 motif at the promoter of these *C. albicans* orthologs, consistent with previous bioinformatics analysis (19), although some of these orthologs are upregulated by activated Put3 in *C. albicans* in our expression data. X-ray crystallography studies of *S. cerevisiae* Put3 DNA binding as well as substitution mutation studies of its binding site reveal that unlike other zinc cluster transcription factors, such as Gal4 and Ppr1, that predominantly bind the CGG DNA half-sites, Put3 binds to the spacer DNA sequence between the half-sites as well (13). Extensive interaction of Put3 with DNA during such binding could render the CGG half-sites dispensable for this transcription factor, facilitating alternative interaction sites in *C. albicans* gene targets beyond its characterized zinc cluster DNA binding motif.

Our findings suggest that *PUT1* and *PUT2* may be under direct and indirect regulation of Put3, respectively, in *C. albicans*, unlike in *S. cerevisiae*, where Put3 regulates both genes through direct DNA interactions (4). As revealed by our RNA-seq, transcription profiling, and ChIP-chip data, Put3 activation may also upregulate ribosome biogenesis genes and carboxylic acid metabolic process genes, as well as genes that are implicated in filamentous growth, the response to stress, RNA metabolic processes, and cellular protein modification (see Table S1 in the supplemental material). Put3 appears to exert a regulatory role in ribosome biogenesis, as over 18% of the transcriptionally upregulated genes observed in our microarray transcription profiling data are annotated with ribosome biogenesis function, compared to just over 4% of *C. albicans* genes that are implicated in ribosome biogenesis. We also noted a very strong signal in our *C. albicans* RNA-seq data, with many ribosome biogenesis genes showing moderate upregulation by proline in the absence of glucose, ammonium sulfate, or both (Table 1). Ribosome biogenesis genes were not upregulated by proline when both glucose and ammonium sulfate were present in the media (Table 1). Such a role has not been previously reported for *S. cerevisiae* Put3. It will be necessary to carry out phenotypic studies to further clarify the role of *C. albicans* Put3 in these pathways and to investigate whether *S. cerevisiae* Put3 plays a similar role. Such investigations may further strengthen a proposed model of functional conservation for Put3 function between the two species and/or could illuminate cases of rewiring in Put3 function.

Overall our findings show that *C. albicans* can utilize proline as a carbon source, a nitrogen source, and both a carbon and nitrogen source. We also demonstrated that *C. albicans* Put3 regulates proline catabolism, even in the presence of ammonium sulfate, to provide the cells with carbon and nitrogen. These functions are in contrast to its role in *S. cerevisiae*, which can only use proline as a nitrogen source. Moreover, Put3 does not possess the ability to effectively activate the proline catabolic pathway in the presence of a rich nitrogen source such as ammonium sulfate in *S. cerevisiae*. Notably, Put3 proline catabolism molecular function is generally conserved between *C. albicans* and *S. cerevisiae*, but their specific catabolic pathways have diverged.

## MATERIALS AND METHODS

### Strains, media, plasmids, and transformation. (i) *Candida albicans*.

The SCPUT3GAD1A strain was constructed as described previously (8). To obtain the *put3* null mutant in the SN95 background strain, one *PUT3* allele was replaced by transformation with the *HIS1* marker and the other allele with the *ARG4* marker. Oligonucleotides used for the *put3* null mutant construction were amplified from plasmids pFA-HIS1 for the first allele knockout and pFA-ARG4 for the second allele knockout (20) using the respective forward and reverse primers PUT3_Marker_KO_F, which contains 97 nucleotides corresponding to the sequence just before the *PUT3* start codon, and PUT3_Marker_KO_R, which contains 95 nucleotides corresponding to the sequence just after the *PUT3* stop codon. Deletion of the first allele was confirmed by PCR using the forward primer PUT3_KO_Check_F, which binds upstream of the *PUT3* gene, and the reverse primer PUT3_KO_Check_R, which binds downstream of the gene. Deletion of the second allele was confirmed by PCR using two primer pairs: (i) forward primer PUT3_KO_Check_F, which binds upstream of the *PUT3* gene, and reverse primer FT-H2, which binds upstream of the *HIS1* gene (*HIS1* promoter region), and (ii) forward primer FT-U3, which binds inside the *HIS1* gene, and reverse primer PUT3_KO_Check_R, which binds downstream of *PUT3*. The *put3* null mutants were confirmed using the forward primer PUT3_KO_Check_Internal_F and the reverse primer PUT3_KO_Check_Internal_R, which both bind inside the *PUT3* gene; no band is expected for *put3* null mutants for this primer pair. Oligonucleotides used for transformation in this study are presented in Table S1.

10.1128/mSphere.00354-17.1 TABLE S1  Oligonucleotides used for DNA amplification in this study. Download TABLE S1, PDF file, 0.1 MB. Copyright © 2017 Tebung et al. 2017 Tebung et al. This content is distributed under the terms of the Creative Commons Attribution 4.0 International license.

Standard procedures for *C. albicans* cell growth and transformation (21) were followed. *C. albicans* strains for transformation, ChIP-chip analyses, and transcriptional profiling experiments were cultured in YPD (1% [wt/vol] yeast extract, 2% [wt/vol] peptone, 2% [wt/vol] dextrose). Yeast nitrogen base medium (YNB) at 6.8 mg/ml supplemented with glucose (20 mg/ml) or proline (15.3 mg/ml) was used for phenotypic studies testing for the ability of the *put3* null mutant to utilize proline as a carbon source. YNB without ammonium sulfate at 6.8 mg/ml supplemented with proline (15.3 mg/ml) was used for phenotypic studies testing for the ability of *C. albicans* (as well as *S. cerevisiae*) wild-type and *put3* null mutant strains to utilize proline as the sole source of carbon and nitrogen. Yeast carbon base (YCB) medium at 11.7 mg/ml supplemented with ammonium sulfate (5 mg/ml) or proline (8.7 mg/ml) was used for phenotypic studies testing for the ability of the *put3* null mutant to utilize proline as a nitrogen source. The *put3* null mutant strains were also cultured in control media (YNB at 6.7 mg/ml without a carbon supplement, YCB at 11.7 mg/ml without a nitrogen source, and YPD).

### (ii) *Saccharomyces cerevisiae*.

*S. cerevisiae* strains were routinely cultured in YPD medium with 40 mg/liter uridine at 30°C. Standard genetic procedures were used for mating of *S. cerevisiae* strains, selection of diploids, induction of meiosis, and tetrad dissection (22). *S. cerevisiae* transformation was carried out by the lithium acetate procedure (23). *PUT3* was replaced by the one-step gene disruption procedure. The *KANMAX4* marker was amplified by PCR of the *Put3* null mutant from the *S. cerevisiae* knockout (YKO) deletion collection using the forward primer *PUT3*-F and reverse primer *PUT3*-R, which provided homology to the flanking regions of the relevant *KANMX4* insert (24). The *KANMAX4* marker was transformed into the prototrophic strain RO-1A (WT-Sacc), and transformants were selected on YPD using Geneticin (G418). Successful deletion of *PUT3* was further confirmed by PCR using two primer pairs: (i) forward primer *PUT3*-F, which binds upstream of the *PUT3* gene, and reverse primer kanB-R, which binds inside the *KANMAX4* gene, and (ii) forward and reverse internal primers *PUT3*-In-F and *PUT3*-In-R, respectively, which bind inside the *PUT3* gene. Oligonucleotides used for transformation in this study are presented in Table S1.

### Proline utilization assays. (i) *Candida albicans*.

The wild-type (WT) strain SC5314, the *put3* null mutant strain ΔΔput3, the Put3 gain-of-function (Put3-GOF) strain SCPUT3GAD1A, and the *ppr1* null mutant strain ΔΔppr1 were each cultured in 10 ml of each of the following media in 50-ml Falcon tubes for 3 days at 30°C with shaking at 220 rpm: 6.8 mg/ml YNB, YNB plus proline (15.3 mg/ml), YNB plus glucose (20 mg/ml), YPD, 11.7 mg/ml YCB (Sigma-Aldrich), YCB plus proline (8.7 mg/ml), and YCB plus ammonium sulfate (5 mg/ml). The *put3* null mutant strain and SC5314 (wild-type strain) were each also cultured for 6 days as described above in the following media: 6.8 mg/ml YNB without ammonium sulfate and YNB plus proline (15.3 mg/ml). Optical density at 600 nm (OD_600_) data were collected every 24 h throughout each incubation period.

### (ii) *Saccharomyces cerevisiae*.

Overnight cultures of the *S. cerevisiae* prototroph RO-1A and the *S. cerevisiae put3* null mutant grown in YPD at 30°C were washed twice in water, adjusted to an OD_600_ of 0.1, and then incubated at 30°C in 10 ml of each of the following media: 6.8 mg/ml YNB, YNB plus proline (15.3 mg/ml), YNB plus glucose (20 mg/ml), YPD, 11.7 mg/ml YCB (Sigma-Aldrich), YCB plus proline (8.7 mg/ml), YCB plus ammonium sulfate (5 mg/ml), 6.8 mg/ml yeast nitrogen base (YNB) without ammonium sulfate, and YNB without ammonium sulfate plus proline (15.3 mg/ml). OD_600_ data were collected every 24 h for up to 3 days or 4 days for cultures in YNB without ammonium sulfate containing proline or no proline.

### Transcriptional profiling experiments.

Transcriptional profiling experiments were carried out as described previously (6), with a few modifications. Briefly, experiments were performed for strain SCPUT3GAD1A (Put3 gain-of-function mutant) compared with the background wild-type strain SC5314. Single colonies of each strain were each inoculated into 10 ml YPD and incubated overnight at 30°C on a 220-rpm shaker. The overnight cultures were diluted to an OD_600_ of 0.1 in 50 ml YPD and grown to an OD_600_ of 0.8. Total RNA was extracted using the Qiagen RNeasy minikit protocol, and RNA quantity was determined using a NanoQuant machine. For direct dye incorporation, 20 μg of total RNA from each sample was reverse transcribed using oligo(dT)23VN and Superscript III reverse transcriptase (Invitrogen) in the presence of Cy3 or Cy5; dye swaps were employed for each sample. Template RNA was eliminated from the synthesized cDNA by simultaneously adding RNase A (Sigma) to a final concentration of 0.05 mg/ml and 0.05 U/µl RNase H (New England Biolabs) to each sample and then incubating the mixture for 30 min at 37°C before purifying the labeled cDNA with a QIAquick PCR purification kit (Qiagen). Hybridization, washing, scanning, and normalization were performed as described previously (25), with the following exceptions. Scanning was carried out using an Axon GenePix 4000B microarray scanner, and data analyses and normalizations were done using GenePix data analysis software. The scanning settings were 635 nm for Cy5 and 532 nm for Cy3. The median of ratios of mutant Cy5-tagged to nontagged Cy3 or mutant Cy3-tagged to nontagged Cy5 values were statistically analyzed in the MultiExperiment Viewer (MeV) software using a *P* value cutoff at 0.05. Positive significant genes (upregulated genes) were candidates for Put3 regulation.

### ChIP-chip.

ChIP-chip experiments were performed as described previously (6) with minor changes. Briefly, the SCPUT3GAD1A strain containing the chromosomally inserted Put3-HA fusion and the background strain SC5314 (untagged) were cultured to an OD_600_ of 0.6 in 50 ml of YPD. Cross-linking for each 50-ml culture was carried out in 1.5 ml of 37% formaldehyde for 30 min, and then ChIP was performed as described previously (6). ChIP DNA extracted from tagged strains was labeled with Cy5 dye, ChIP DNA from untagged strain SC5314 was labeled with Cy3 dye, and the samples were then cohybridized to Agilent 8X15K whole-genome arrays containing 14490 60-mer intergenic and intragenic oligonucleotide probes. Microarray hybridization, washing, scanning, and normalization were performed as described previously (25) with the following modifications: the Axon GenePix 4000B microarray scanner was used to perform scanning, and GenePix data analysis software and Multiexperiment Viewer (MeV) software were used to analyze and normalize data; a 0.05 *P* value cutoff was used for MeV analyses. The scanning settings used were 635 nm for Cy5 and 532 nm for Cy3. The log of ratios of Cy5 to Cy3 (635 nm/532 nm) with a cutoff of at least 1.5 for each spot was considered to be an indicator of significant Put3 binding.

### RNA-seq.

Single colonies of the *C. albicans* wild-type strain SC5314 and the *put3* null mutant strain were each inoculated into 10 ml YPD and incubated overnight at 30°C on a 220-rpm shaker. The overnight cultures were diluted to OD_600_ of 0.1 in 10 ml and grown to an OD_600_ of between 0.8 and 1.3 in various media at 30°C with shaking at 220 rpm. SC5314 was cultured in YNB plus ammonium sulfate, glucose, and proline (YNGP_SC), YNB plus ammonium sulfate and glucose (YNG_SC), YNB plus ammonium sulfate and proline (YNP_SC), YNB plus glucose and proline (YGP_SC), and YNB plus proline (YP_SC). The *put3* null mutant strain was cultured in YNB plus ammonium sulfate, glucose, and proline (YNGP_ΔΔPut3) and YNB plus ammonium sulfate and glucose (YNG_ΔΔPut3). YNB (6.8 mg/ml), proline (15.3 mg/ml), glucose (20 mg/ml), and ammonium sulfate (5 mg/ml) were used when required. Total RNA was extracted using the Qiagen RNeasy minikit protocol, and RNA quality and quantity were determined using an Agilent bioanalyzer. Sequencing of extracted RNA samples was carried out at the Quebec Genome Innovation Center located at McGill University using an Illumina miSEQ sequencing platform. Each RNA-seq data file was postprocessed to correct read sequences (26), trim adapters (27), and remove rRNA reads (28). The C. albicans_SC5314_Assembly22 ORF/gene coding sequences (SC5314_V22) were downloaded from the CGD website (29). A PERL script was written to create a gene/open reading frame ID and description file. The reads were then mapped to the SC5314_V22 sequences (30) to produce raw counts and TPM (transcripts per million) values. Raw counts and TPM values were annotated with gene descriptions using a PERL script and imported to an Excel spreadsheet for further analysis. Expression ratios of mutants versus controls in each experiment were calculated to identify proline-dependent changes in gene expression. To minimize false positives, only gene expression values of 0.5 or above in control data were considered in the calculation of the ratios of experiments versus controls.

### Bioinformatics.

The CGD tool “Go Term Finder” (http://www.candidagenome.org/cgi-bin/GO/goTermFinder) (31) was used for Gene Ontology analyses. Fungal BLAST analysis of *C. albicans* Put3 was performed using the Saccharomyces Genome Database (SGD) fungal BLAST tool (http://yeastgenome.org/blast-fungal) (32). Protein sequences were aligned using the SIM Alignment tool (http://web.expasy.org/sim/) (33), and graphical representation of protein alignment was generated using the LALNVIEW program (34). Put3 alignments throughout the ascomycete lineage were carried out using Fungal Orthogroups Repository (https://portals.broadinstitute.org/regev/orthogroups/) (35). Color coding of amino acid classes in the Put3 and ortholog sequences was done using an online tool, the Sequence Manipulation Suite (36). A Newick file for Put3 yeast phylogeny was generated using Fungal Orthogroups Repository (35) and then modified and used to generate the Put3 phylogenetic tree using an online Newick Viewer, T-REX (37).

### Data availability.

ChIP-chip and transcription profiling (microarray) data can be found in Table S2, and RNA-seq data can be found in Table S3.

10.1128/mSphere.00354-17.2 TABLE S2  ChIP-chip and transcription profiling data for Put3-HA (SCPUT3GAD1A) versus SC5314 in *C. albicans*. Download TABLE S2, XLSX file, 1.6 MB. Copyright © 2017 Tebung et al. 2017 Tebung et al. This content is distributed under the terms of the Creative Commons Attribution 4.0 International license.

10.1128/mSphere.00354-17.3 TABLE S3  RNA-seq data showing proline-induced genes in *C. albicans*. Download TABLE S3, XLSX file, 2.6 MB. Copyright © 2017 Tebung et al. 2017 Tebung et al. This content is distributed under the terms of the Creative Commons Attribution 4.0 International license.
