# Supplementary material for: Put3 Positively Regulates Proline Utilization in Candida albicans
Source: mSphere. 2017 Dec 13;2(6):e00354-17. doi: 10.1128/mSphere.00354-17 (PMC5729217; doi:10.1128/mSphere.00354-17)
Supplement: TABLE S1 [file sph006172429st1.pdf]

Table S1: Oligonucleotides used for DNA amplification in this study.

**Name**	**Sequence (5' - 3')**
PUT3_Marker_KO_F	CATTCCGAACAAGGTATTAGAAATAATTATTTTCTATCAACC AACACCCACATAAATCATTCCTTCATTTACTTATATATAATC CGATTCTTGTACAGAAGCTTCGTACGCTGCAGGTC
PUT3_Marker_KO_R	GTTTTTGTAATATATTGTATTATATAGAAAATTTTATTACCAT CACAGAATAAATGTACAGACATAAATATATATTTGCCTCAC TCCCGCACAATTCTGATATCATCGATGAATTCGAG
PUT3_KO_Check_F	CGTTGTGCGAAACTACCAAA
PUT3_KO_Check_R	ACAGGCCAATGAGAATACGC
PUT3_KO_Check_Internal_F	AAGAGCCTTCGGAGGAAAAG
PUT3_KO_Check_Internal_R	TCGGGACTTTTTGTTTGGAG
FT-U3	TATAGGTCTTAGTGTTGACTGT
FT-H2	CAACGAAATGGCCTCCCCTACCACAG
ScPUT3-F	ACATCCACCAGGTGCTTGATGA
ScPUT3-R	TGGACAGAAGGTGATAGCGACA
ScPUT3-In-F	GGCAACATTCCAGCAGGCTTAA
ScPUT3-In-R	TCAAATATAGGAGCCGCCGGAG
kanC-F	TGATTTTGATGACGAGCGTAAT
kanB-R	CTGCAGCGAGGAGCCGTAAT
